# A survey of health problems of Nepalese female migrants workers in the Middle-East and Malaysia

**DOI:** 10.1186/s12914-018-0145-7

**Published:** 2018-01-18

**Authors:** Padam Simkhada, Edwin van Teijlingen, Manju Gurung, Sharada P. Wasti

**Affiliations:** 1 Public Health Institute, Liverpool John Moores University, Henry Cotton Building, 15-21 Webster Street, Liverpool, L3 2ET UK; 2 Manmohan Memorial Institute of Health Sciences, Tribhuvan University, Kirtipur, Nepal; 3 Nobel College, Pokhara University, Pokhara, Nepal; 4 Faculty of Health & Social Sciences, Bournemouth University, Bournemouth, UK; 5 Pourakhi Nepal, Makhamali Marg, Maharajgunj, Kathmandu, Nepal; 6 Institute for Reproductive Health, Georgetown University, Washington, DC, USA

**Keywords:** Health problems, Migration, Exploitation, South Asia, Nepal, Women, Gulf countries, Malaysia

## Abstract

**Background:**

Nepal is a key supplier of labour for countries in the Middle East, India and Malaysia. As many more men than women leave Nepal to work abroad, female migrant workers are a minority and very much under-researched. The aim of the study was to explore the health problems of female Nepalese migrants working in the Middle-East and Malaysia.

**Methods:**

The study was conducted among 1010 women who were registered as migrant returnees at an organisation called Pourakhi Nepal. Secondary data were extracted from the records of the organisation covering the five-year period of July 2009 to July 2014.

**Results:**

The 1010 participants were aged 14 to 51 with a median age of 31 (IQR: 38-25) years. A quarter of respondents (24%) reported having experienced health problems while in the country of employment. Fever, severe illness and accidents were the most common health problems reported. Working for unlimited periods of time and not being able to change one’s place of work were independently associated with a greater likelihood of health problems. Logistic regression shows that migrant women who are illiterate [OR = 1.56, 95% CI: 1.02 to 2.38, *p* = 0.042], who had changed their workplace [OR = 1.63, 95% CI: 1.14 to 2.32, *p* = 0.007], who worked unlimited periods of time [OR = 1.64, 95% CI: 1.44 to 1.93, *p* = 0.020], had been severely maltreated or tortured in the workplace [OR = 1.84, 95% CI: 1.15 to 2.92, *p* = 0.010], were not being paid on time [OR = 2.38, 95% CI: 1.60 to 3.55, *p* = 0.038] and migrant women who had family problems at home [OR = 3.48, CI 95%: 1.22 to 9.98, *p* = 0.020] were significantly associated with health problems in their host country in the Middle East.

**Conclusion:**

Female migrant workers face various work-related health risks, which are often related to exploitation. The Government of Nepal should initiate awareness campaigns about health risks and rights in relation to health care services in the host countries. Recruiting agencies/employers should provide information on health risks and training for preventive measures. Raising awareness among female migrant workers can make a change in their working lives.

**Electronic supplementary material:**

The online version of this article (10.1186/s12914-018-0145-7) contains supplementary material, which is available to authorized users.

## Background

Globalisation of markets and labour supply has added impetus to the growing mobility of people working abroad. In Asia the movement of labour is an important and enduring phenomenon associated with economic growth and development since it eases skill imbalances in labour markets and provides benefits for both sending and receiving countries [1]. Millions of people who are unable to get jobs in low-income countries leave their country for better paid employment opportunities abroad [2]. At the same time, the number of low-skilled women migrating through illegal, undocumented, or clandestine routes has increased, which makes them vulnerable in their host country [3]. Most migrant workers exist at the bottom of the economic hierarchy; they work hard, often sending money home to support their families. However, these positive aspects of migration come at a high price. The negative factors associated with migrant labour include injury and illness, as well as poor provision of health care [4]. While migration in and of itself is not necessarily a risk to health, the conditions surrounding the process can increase health vulnerabilities [5]. In extreme situations, migrants are forced to return home because of ill-health, chronic or terminal illnesses, and often end up unable to work or die back in their home country [6, 7].

Women currently account for nearly 50% of all migrants worldwide. An estimated 2.5 million Nepalese women are currently working outside of Nepal, in countries that include Malaysia and the Middle East. The latter is sometimes referred to as the Gulf Corporation Council (GCC) countries, including, amongst others, the United Arab Emirates (UAE), Saudi Arabia, Qatar, and Bahrain. An estimated 90% of female Nepalese migrant workers are undocumented; and the number of female migrants is highest in Saudi Arabia, followed by Kuwait, Qatar, Oman and the UAE [8]. Foreign labour migration has become both an opportunity and a challenge for many Nepalese women. Female migrant workers comprise 11% of the contributors of remittances, whilst all remittances make up 32% of Nepal’s Gross Domestic Product [9]. The reasons behind migration are very similar in Nepal to other parts of the world, which include: poverty, limited employment opportunities and deteriorating agricultural productivity at home, as well as armed conflict in the recent past [10].

Migration has created health and health care challenges in both countries of origin and destination. Migrants can face serious access to health care due to discrimination, language and cultural barriers, their uncertain legal position, and their low socio-economic status [11]. Moreover, more than 500 Nepalese migrant workers have died in the Gulf region in workplace-related accidents and/or due to poor labour conditions (e.g. lack of safety standards and formal labour relations) and mental illness (including suicide). These factors may have contributed to higher mortality rates for Nepalese migrants in this region. Work-related injuries, deaths from various causes, and suicides are relatively common in GCC countries [12]. It is estimated that two Asians die per day on Dubai construction sites and one suicide occurs every 4 days [13]. There have also been unexplained deaths of Nepalese migrants who receive low wages and live in poor housing, eat unhealthy food, and have difficulty accessing health services and who may have been exposed to other risk factors that promote poor health [14]. These migrants often return home less healthy than when they left. Health care services are often expensive for migrants and sometimes migrants even have to pay a deposit before they will receive health services.

The working/living conditions of women in foreign employment to Gulf countries is a topic regularly covered in Nepali mass media, but despite this media coverage there has been very little academic research on the topic. The aim of the study is to explore health issues of Nepalese women migrants working in Gulf countries.

## Methods

The study was based on secondary analysis [15] of information collected from 1010 female returnee migrants registered with an emergency shelter in the period from July 2009 to July 2014 with Pourakhi, a NGO (Non-Governmental Organization) in Nepal. A counsellor from Pourakhi first collect the details information from women using an Information Form (see Additional file 1) once they returned to Nepal. Study respondents were interviewed (in private) using this Client Information Form at the NGO which provides shelter to women returnee migrants who seek its support. Our research team then extracted the required information from the Client Information Form using a standard questionnaire (see Additional file 2). This questionnaire was pretested to ensure we collected the relevant information consistently. Most of the study questions in the questionnaire were adopted from previous migrants’ surveys which had been used in similar settings, hence the questionnaire was culturally appropriate for women in a Nepali context, and this process ensured that the questionnaire was, to a degree, valid and reliable. The primary outcome variable in the analysis was self-reported health problems during the women’s stay abroad. The questionnaire established whether women answered ‘yes’ or ‘no’ to the question asked by Pourakhi staff: We asked ‘did you have any health related problem while you were abroad?’

The analysis included descriptive statistics, including chi-square tests and multiple logistic regression analyses - the latter to find any independent factors associated with health problems in foreign employment. We have used the multiple regression analysis to determine the health issues among migrants’ women. The dependent variable was ‘health problem’, independent variables were socio-demographic, migration and other health condition related variables where dependent variable was coded health problem 0 = no and 1 = yes. After the initial univariate analysis only significant variables (95% CI less than 0.05 *p* value) were inserted into multivariate logistic regression and findings are presented in the result section. SPSS version 22 was used for the analysis. Data were double entered and cross-checked, in order to enhance reliability.

Ethical approval was obtained from Nepal Health Research Council (150/2012) and verbal consent was taken from respondents who were assured regarding anonymity and confidentiality and no names were recorded in the secondary data set for analysis.

## Results

### Socio-demographic characteristics

A total of 1010 female respondents were registered with Pourakhi, their ages ranging from 14 to 51, with a median age of 31 years. A large minority of respondents 435 (43.1%) belonged to the 26-to-35 age group, followed by those 36 and over (Table 1).

**Table 1:** Socio-demographic characteristics of female respondents

Variables	Number	Percentage
Age group (years)		
≤ 25	259	25.6
26- 35	435	43.1
≥ 36	316	31.3
Caste/Ethnicity		
Brahmin/Chhetri	194	19.2
Indigenous group	434	43.0
Dalit/Minor group	243	24.1
Terai origin (Madhesi/Tharu)	139	13.8
Region of origin		
Eastern development region	402	39.9
Central development region	217	21.5
Western development region	236	23.3
Mid-western development region	141	14.0
Far-western development region	14	1.3
Education Status		
Illiterate	378	37.4
Primary	426	42.2
School Leaving Certificate & higher	206	20.4
Marital Status		
Never married	242	24.0
Married	489	48.4
Divorced/Separated	209	20.7
Widowed	70	6.9
Country of foreign employment		
Kuwait	571	56.5
Saudi Arabia	270	26.7
United Arab Emirates	54	5.3
Lebanon	68	6.7
Other	47	4.7
Passport details		
Own name	965	95.5
Illegitimate travel documents	45	4.5
Types of work		
Domestic	985	97.9
Company	21	2.1
Methods of return home		
Released by ‘owner’/ employer	572	56.6
Escaped from work place	224	22.2
Returned by police	109	10.8
Rescued by agent	56	5.5
Rescued by Nepal embassy/Non Residential Nepali	49	4.9

The caste/ethnic distribution shows that 43.0% were indigenous women, followed by Dalit (lower caste) women (24.1%), whereas (higher caste) Brahmin and Chhetri are the least numerous (19.2%) group. The findings show that nearly half (40%) of the women were from the eastern development region, followed by 23.3% from the western development region. In all, 37.4% were illiterate; a slightly higher percentage had completed primary school as their highest level of education (42.2%), whilst the smallest group consisted of those who had achieved secondary/higher education (20.4%). The descriptive analysis further shows that 48.4% of female migrants were married, followed by unmarried (24.0%), divorced/separated (20.7%) and widowed (6.9%). All did domestic work in the country of foreign employment. More than half (56.5%) of female workers had worked in Kuwait, followed by Saudi Arabia (26.7%), Lebanon (6.7%), UAE (6.7%) and 4.7% in other countries of the Middle-East. Around 5 % of the women had used illegitimate documents to travel to GCC countries as a migrant worker (Table 2).

**Table 2:** Health status & problems faced among migrant women in the workplace

Variables	Number	Percentage
Health problem at work	242	24.0
Failed medical test	35	3.5
Abuse at workplace	413	40.9
Accident at workplace	12	1.2
Physical harm	112	11.1
Had mental health problem	88	8.7
Torture at work	311	30.8
Pregnancy at work place	31	3.1
Cause of pregnancy		
• Sexual abuse	16	50.1
• Consensual relation	15	49.9
Received health services at workplace	130	12.9

Nearly a quarter (24.0%) of women had experienced health problems in their destination country. Many had suffered abuse at the workplace (40.9%), whilst ‘only’ 12 (1.2%) reported to have suffered work-related injuries. Around 9 % of respondents reported that they had mental health problems, followed by 11.1% having faced physical harm, and 30.8% who faced torture or maltreatment at the workplace; 3.1% migrant women were reported to have fallen pregnant in the workplace, half of whom (50.1%) as a consequence of rape/sexual abuse. A further 15 of the pregnant women (49.9%) reported consensual sexual relationships (a conduct strictly forbidden in Muslim countries). The study shows that only 12.9% respondents reported that that they had received attended health services after falling ill whilst in domestic work abroad.

### Health problems among the migrant women

Table 3 analyses health problems by using univariate and multivariate logistic regression analysis. In the univariate analysis, significant difference was observed between the migrant women who had health problems and those who did not, regarding educational status, received pre-departure training, change of work place, leave provision, working hours, experiencing severe maltreatment or torture at their work place, not getting paid, or had family problems at home (< 0.05).

**Table 3:** Factors influencing health problems in logistic regression analysis

Variables	Health problems				Univariate			Multivariate		
	Yes	%	No	%	OR		P	OR		P
Age group										
Age 31 and below	124	23.9	395	76.1	0.944	0.94 to 0.82	0.427			
Above 31 years	114	23.2	377	76.8						
Education status										
Illiterate	93	24,6	285	75.4	1.71	1.48 to 1.98	<0.001	1.56	1.02 to 2.38	0.042
Literate	145	22.9	487	77.1						
Marital status										
Unmarried	47	19.4	195	80.6	0.71	0.49 to 1.01	0.059			
Other	191	24.9	577	75.1						
Received pre-departure training	57	39.0	89	61.0	2.27	1.57 to 3.29	<0.001			
Change of work place	55	19.1	233	80.9	1.44	1.03 to 2.10	0.034	1.63	1.14 to 2.32	0.007
Leave provision	20	27.8	52	72.2	1.77	1.06 to 2.94	0.028			
Working hours										
Unlimited	93	29.0	228	71.1	1.59	1.18 to 2.14	0.003	1.64	1.44 to 1.93	0.020
12 to 18 hours	145	21.0	544	79.0						
Torture at workplace	51	16.4	260	83.6	1.75	1.25 to 2.45	0.001	1.84	1.15 to 2.92	0.010
Unpaid for some of the work	37	15.5	198	84.3	1.85	1.26 to 2.72	0.002	2.38	1.60 to 3.55	0.038
Had home loan	7	21.9	25	78.1	0.89	0.50 to 1.55	0.67			
Had fixed salary	130	20.2	512	79.8	1.31	0.96 to 1.79	0.086			
Salary on time	103	19.8	417	80.2	1.28	0.94 to 1.73	0.12			
Family problem at home	39	90.7	4	9.3	3.18	1.13 to 9.0	0.029	3.48	1.22 to 9.98	0.020

Multivariate binary logistic regression models were constructed and analysed with the occurrence of health problems as the dependent variable: all significant bivariate logistic regression analysis findings (Table 3) show that female migrants who were illiterate were 1.56 times more likely to be linked to the occurrence of health problems, when compared to literate migrant women [OR = 1.56, 95% CI: 1.02 to 2.38, *p* = 0.042]. The odds of the occurrence of health problems among the workers who changed between workplaces in the country of migration were 1.6 times more likely to have health problems, compared to those who continue to work at the same place [OR = 1.63, 95% CI: 1.14 to 2.32, *p* = 0.007]. Similarly, those working for unlimited periods of time (beyond time agreed in their contract, and without added remuneration) were 1.64 times more likely to have health problems compared to those who worked for a ‘normal’ 12–18 h a day [OR = 1.64, 95% CI: 1.44 to 1.93, *p* = 0.020]. The odds for subsequent health problems among the workers who had been tortured or maltreated in the workplace was 1.84 times greater than those had not faced any such workplace torture or maltreatment [OR = 1.84, 95% CI: 1.15 to 2.92, *p* = 0.010]. Similarly, the odds of health problems among the migrant workers who had not been paid salary for added work, were 2.38 times greater than those who were paid as per contract [OR = 2.38, 95% CI: 1.60 to 3.55, *p* = 0.038]. Moreover, migrant women with family problems at home were 3.48 times more likely to have health problems at the workplace in Middle-East countries compared to those without family problems [OR = 3.48, CI 95%: 1.22 to 9.98, *p* = 0.020].

However, study findings did not find the statistical significance regarding health problems during their employment stay in Middle-East countries between different age groups (*p* = 0.427), marital status (*p* = 0.059), having had home loan (*p* = 0.67), having a fixed salary at their work place (*p* = 0.086), and receiving salary on time (0.12), in the bivariate logistic regression analyses.

## Discussion

Foreign employment has been attracting numerous Nepalese men and women mainly because it provides them with the opportunity of financial independence [16]. According to the status report of labour migration for Nepal, Malaysia received most Nepalese labour migrants (40.9%), followed by Saudi Arabia (22.9%), Qatar (20.3%), UAE (11.2%) and Kuwait (2.1%), which are all major destination countries for foreign employment from various countries. The proportion of male labour migrants compared with females is very high in Malaysia, Qatar and Kuwait. This is different from the global scenario where women represent approximately half of all international migrants [17]. However, there has been significant increase in the number of permits acquired by Nepalese women between the periods of 2008/9 to 2013/14 [18, 19]. In our study, the distribution of the migrant workers in terms of destination country was Kuwait (56.5%), Saudi Arabia (26.6%), Lebanon (6.5%) and UAE (5.3%) which is different from the results of a similar study conducted in Nepal that showed the distribution as Qatar (50.2%) followed by Saudi Arabia (33.6%) and UAE (16.2%). Nevertheless, we have only assessed the female migrant workers in this study and not included the male migrant workers which could have caused the differences in results of these two studies. A UN report showed that Nepalese women - mostly younger than 25 – commonly worked in the Gulf countries [19]. In contrast to this, Joshi et al. reported that 53.4% of the total migrant workers were in the age group of 26-35 years old [12]. Our study also revealed that only a quarter of the total women (26.5%) were below 25 years of age and 42.3% were between 26 and 35 years with a median age of 31 years. The majority of the study participants came from indigenous groups (45.4%), and ethnicity is linked with socioeconomic status in Nepal, the indigenous and Dalit groups having the lowest status [12]. The rising trend, of trauma-related sickness among indigenous communities might be due to their lower socio-economic status. Such results illustrate the strong nature of poverty, which can even break social traditions and gender restrictions imposed on women [18]. A majority of the women in our study (42.2%) had obtained only a primary-school level of education, while 37.4% were illiterate. Similarly in the study by Joshi and colleagues, Nepalese migrants working in Gulf countries had an education level below secondary (10th grade) [12]. Illiteracy or inadequate education could have played a greater role in the heightened vulnerability of these women to poverty and other negative health problems.

Female workers from Sri Lanka also encounter problems in the Middle East including limited freedom of movement, lack of social protection, poor living and working conditions, harassment, violence and mental illness [20]. Migration has created challenges in both countries of origin and destination. The risk of serious health problems are intensified due to discrimination, language and cultural barriers, a weak legal position and low-socioeconomic status [12, 21]. Moreover, female migrants were not given enough time by their employers to rest and recover from an illness, accident, injury, or surgery [22]. Nevertheless, the Government of Nepal appears to be more concerned with remittances earned from workers abroad, than with their health and wellbeing in the destination countries [23–25].

Our study found that about a quarter (24%) of respondents had health problems, while another recent study of Nepalese migrants in Gulf countries found that nearly half (56.6%) of the workers had experienced health problems, such as headaches and fever, respiratory problems, musculoskeletal problems, gastro-intestinal illness, injuries/poisoning and ‘accidents’ [12]. The higher percentage in the latter study may have been due to involvement in different occupations, especially construction work, agriculture and farming. In contrast, in our study all were employed as domestic workers. A recent review found that the key risk factors in migrant workers from Nepal are: (i) sexual risk-taking behaviour; (ii) occupational injuries and hazards; and (iii) lifestyle changes, however the majority of the 18 included studies focused on male migrant workers [26].

Early detection and treatment of health problems might prevent adverse health outcomes among the international migrants. This could be achieved through increased access to, and affordability of health care in the destination country as well as in the country of origin [27]. Nearly a quarter of migrants in our study reported experiencing health problems ranging from fever to severe illnesses and ‘accidents’ (or physical injuries incurred in the workplace which were labelled as accidents) while working abroad. As the data for our study was based on the information collected by Pourakhi Nepal, we do not have details on the specific types of health problems faced by the study participants. The study found that nearly one-third (30%) of respondents reported torture or maltreatment at work which is extremely high. One possible explanation is that our population is not all Nepali women who worked abroad, but a sub-sample of those who were rescued, came back with problems or due to problems, and hence sought support from the charity Pourakhi.

Results from multivariate analysis shows that long working hours, workplace change, illiterate workers, delays in salary being paid, and ‘torture at their work place’ (presumably severe beatings, burnings, etc.) at the hands of employers, were significantly associated with likelihood of occurrence of concurrent and subsequent health problems, adjusting for other possible confounding factors. The findings of our study are consistent with the findings from the United States [28], where working for very long hours in jobs is associated with health hazards, compared to jobs without prolonged overtime: elsewhere it has been reported that excessive working hours result in ‘accidents’ and injuries at work [29]. Family problems at home, torture at work as well as being unpaid for some of the work were associated to the occurrence of health problems among the migrant workers included in our study. Similar findings were also reported in Sri Lankan female migrant workers in Middle-Eastern countries and Filipino female migrant workers in Hong Kong [30, 31]. However, the health of female migrants is not always poorer in the receiving countries compared with their home countries. Some studies suggest that migrants can have a positive health experience when they move from a low-income to a high-income country [32–34]. Further research is needed to understand the in-depth dynamics of health among Nepalese male and female migrant workers to understand the health and their lifestyle working abroad.

### Strength and limitations of the study

This is first kind of study which has accessed women returnee migrants from the Middle-East, in Nepal. This study was limited however by only accessing registered returnee women migrants in Pourakhi [a national-level NGO], which may limit its generalisability. It is possible that Pourakhi as a centre in Kathmandu attracted women who needed specific help or were unable to travel directly to their homes, and thus our sample may have be biased towards returnees with health and social problems. Since we used data from subjective reports, often without verification, the reliability and validity of the information collected should be treated with caution. Hence, we recommend that further systematic study in this extremely important research topic in international health should be undertaken.

## Conclusions

Migration can have a profound effect on the health and well-being of some individuals. It appears that a significant proportion of female migrant workers in the Middle-East experience some sort of health problems, as a result of harsh living and working conditions. Nearly a quarter of female migrants in our sample had experienced health problems such as fevers, severe illnesses and physically traumatic ‘accidents’, which appeared to be common health problems experienced during their working abroad. Those who worked for unlimited periods of time (beyond a 12-h day), who were illiterate, physically and sexually abused, and even tortured at their work place, and who were denied payment, were factors independently associated with increased likelihood of developing a variety of health problems. It is important, given these findings that all potential migrants must be made aware of potential health risks, and their rights in relation to health care services in the country of foreign employment. It is advised that Middle-East countries should ensure their migration policies and practices should be monitored according to international norms, ensuring both proper working conditions and treatment of employees, and the availability and accessibility of health care services to migrant workers during their stay. The present study shows that only 13% of migrant workers had access to health services during their contracts. The Government of Nepal should also start a dialogue with host countries, recruitment agents/brokers, and moreover should systematically negotiate maximum working hours, sufficient food, minimum salary, health facilities, and security at the workplace: in other words the kind of things we would expect a trade union to do elsewhere. Recruiting agencies and employers should also provide training for preventative health measures for health risks. The proportion of women engaged as migrant workers is still growing, so we need a better understanding of the nature of health aspects as well as an increased capacity to address the health needs of women working abroad.

## Additional files

Additional file 1: Client Information Form. (DOCX 16 kb)

Additional file 2: Questionnaire for data extraction. (DOCX 16 kb)
