# Supplementary material for: A survey of health problems of Nepalese female migrants workers in the Middle-East and Malaysia
Source: BMC Int Health Hum Rights. 2018 Jan 18;18:4. doi: 10.1186/s12914-018-0145-7 (PMC5774120; doi:10.1186/s12914-018-0145-7)
Supplement: Supplementary file 1 — Client Information Form. (DOCX 16 kb) [file 12914_2018_145_MOESM1_ESM.docx]

**POURAKHI NEPAL**

**Case report- Client Information Form**

(Unofficial translation of from Nepali to English)

Date: Referral person/institution:

**General information**

Name of the client:

Gender:

Age:

Address:

Contact no:

Date of birth:

Passport number:

Departure date:

Work permit number:

| Marital status: |  |
| --- | --- |
| Who are there in the family? |  |
| Qualification: |  |
| Family information (Genogram): |  |
| Family relation: |  |
| Destination country: |  |
| Years in foreign employment: |  |
| Reason for foreign employment: |  |
| Mode for foreign employment: |  |
| Legal status while going foreign employment: | - Documented - Undocumented |
| Times for foreign employment: |  |
| Experience during the stay in foreign employment: |  |
| Reason return to Nepal: |  |
| Current status/problem: |  |
| Who is helping the client? |  |
| With whom the client is in contact with (person/friend/institution): |  |
| Sources of the case: | - Family/Friend: - Institution: - Potentiality: - Qualification: - Liquid/ Fixed assets (if any): |
| Services provided by Pourakhi Nepal | - Accommodation - Health services - Legal services - Counseling |
| **Activities done to help the client:** | |
| 1. Accommodation: | Progress of the case:  Social status of the client:  Financial status of the client:  If any further help needed to the client, where do you refer the client?  Reason closing the case: |
| 2. Health services: | Health status of the client:  If any further help needed to the client, where do you refer the client?  Reason closing the case: |
| 3. Legal services | Legal treatment:  Progress of the case:  If any further help needed to the client, where do you refer the client?  Reason closing the case: |
| 4. Psycho-social counseling | How many sessions done for counseling:  Subjects discussed:  Status of the client after counseling:  Techniques applied for counseling:  Result:  Progress of the client:  If any further help needed to the client, where do you refer the client? |
