# Supplementary material for: A survey of health problems of Nepalese female migrants workers in the Middle-East and Malaysia
Source: BMC Int Health Hum Rights. 2018 Jan 18;18:4. doi: 10.1186/s12914-018-0145-7 (PMC5774120; doi:10.1186/s12914-018-0145-7)
Supplement: Supplementary file 2 — Questionnaire for data extraction. (DOCX 16 kb) [file 12914_2018_145_MOESM2_ESM.docx]

**POURAKHI NEPAL**

**Data Extraction Sheet**

Please look the ‘Client Information Form’ and complete the following questions. Please consult to Pourakhi staff if any information is not clear in Client Information Form.

Respondent code:

Age:

Address district:

**Section I: Socio-demographic characteristics**

| S.N | Questions | Options | Remarks |
| --- | --- | --- | --- |
| 1 | What is your caste/ethnicity? | 1.Brahmin/Chhetri  2.Indigeneous  3.Dalit  4.Muslim |  |
| 2 | Geographical location | 1.Mountain  2.Hill  3.Terai |  |
| 3 | Which religion do you follow? | 1.Hindu  2.Buddhism  3.Islam  4.Christian |  |
| 4 | What is your educational status? | 1.Illiterate  2.Literate  3.Primary (1-5)  4.Secondary(6-10)  5.+2 and above |  |
| 5 | Marital status | 1.Married  2.Unmarried  3. Divorced  4.Widow  5. Separated  6.Living together |  |
| 6 | Number of children | 1.No children  2……… children |  |
| 7 | Types of family | 1.Joint family  2.Nuclear family |  |

**Section II: Decision making process and journey**

| 1 | Times for foreign employment | 1.First time  2.Second time  3.Three and more |  |
| --- | --- | --- | --- |
| 2 | Reason for foreign employment | 1.Well being of the family  2.Home loan  3.Children education  4.Unemployment  5.Political conflict  6.Violence  7.Landless  8.Peer influence |  |
| 3 | Mode for foreign employment | 1.Recruitment agency  2.Agency  3.Friend/relatives |  |
| 4 | Pre-departure training | 1.Yes  2.No |  |
| 5 | Pre-departure cost | 1.Didn’t pay  2.Rs………. |  |
| 6 | Information about job before going for foreign employment | 1.Work  2.Salary |  |
| 7 | Legal status while going foreign employment | 1.Undocumented  2.Documented |  |
| 8 | Departure place | 1.TIA  2.India |  |

**Section III: Employment in the destination country**

| 1 | Destination country | 1.Kuwait  2.Saudi Arabia  3.Lebanon  4.Oman  5.Others………….. |  |
| --- | --- | --- | --- |
| 2 | Types of work in destination country | 1.Domestic  2.Company  3Didn’t work |  |
| 3 | Working hour | 1.Unlimited  2.12-18 hours  3.8 hours  4.Didn’t work |  |
| 4 | Duration in foreign employment | 1.Less than 6 months  2.6 months to 1 Year  3.1 Years to 2 Years  4.2 Years to 3 Years  5.Above 3 Years |  |
| 5 | Work place change | 1.Yes  2.No |  |

**Section IV: Problems faced at abroad**

| 1 | Health problems at work | 1.General(fever and seasonal illness)  2. Severe(operation)  3. Accident at work place  4.Nothing |  |
| --- | --- | --- | --- |
| 2 | Health services received | 1.Yes  2.No |  |
| 3 | Health service provider | 1.Employer  2.Recruitment agent  3.Nepal Embassy  4.Self |  |
| 4 | Harassment faced by women | 1.Physical  2.Sexual  3.Mental  4.Verbal |  |
| 5 | Problems faced at work | 1.Workload  2.Unpayment  3.Restriction to contact payment |  |
| 6 | Who are the perpetrators? | 1.Employer  2.Agency  3.Employer’s family member  4.Colleague/Boy friend |  |

**Section V: Repatriation**

| 1 | Medium departure from destination | 1.Employer  2.Embassy of Nepal  3.Agency  4.Escaped  5.Police administration  6.Airport  7.Self  8.Organization |  |
| --- | --- | --- | --- |
| 2 | Reason for return from foreign employment | 1.Illegal status  2.Unpaid salary  3.Torture at work  4.Illness  5.Pregnancy  6.False accused  7.Completion of contract  8.Family problem  9.Voluntary return  10.Medical fail |  |

**Section VI: In-country**

| 1 | Document on arrival | 1.Passport  2.Travel document |  |
| --- | --- | --- | --- |
| 2 | Health condition on arrival | 1.Illness  2.Injury  3.Mental  4.Normal  5.Pregnant  6.With child |  |
| 3 | Causes for Pregnancy | 1.Consensual relation  2. Own Husband in Nepal  3. Sexual abuse |  |
| 4 | Services at shelter home | 1.Accommodation  2. Health care  3. Transportation |  |
| 5 | Days at shelter home | 1.One day  2.Upto one week  3. More than a week  4. Counseling |  |
| 6 | Days at hospital | 1.One day  2.Upto one week  3.More than a week |  |
| 7 | Re-integration of migrant returnee | 1.With family/ relatives  2. Self  3. Long term shelter  4. Friend |  |

**The End!**
